# The Duty of the General Practitioner toward the Specialist1President’s address, read at the thirty-second annual meeting of the Medical Association of Central New York, at Syracuse, October 17, 1899.

**Published:** 1899-11

**Authors:** E. L. Mooney

**Affiliations:** Syracuse, N. Y.


### THE DUTY OF THE GENERAL PRACTITIONER TOWARD THE SPECIALIST.[1]

By E. L. MOONEY, M. D., Syracuse, N. Y.

WHAT general practitioner has not had cases which have puzzled him to such an extent that his diagnosis was far from clear or satisfactory to himself at the time and later found that he was far from right, and what have we done in these cases? We have gone on treating them along the lines we thought best, hoping and trusting that we were right, and anxiously awaiting developments.

It seems to me that in continuing in this uncertain manner we are not doing justice to our patients, to our professional standing, or to our conscience, but instead we should call in consultation a professional brother, who has had greater experience, or who has devoted several years of special study and work in the class of diseases with which our patient is suffering—namely, a specialist. In a great majority of cases the cause of the malady is very plain to him, and if it can be removed, and is, then our patient is either cured or greatly benefited, and we get our share of praise from the patient and we are also satisfied that we have done our duty to the patient, the specialist, and ourselves. I will give the history of a few cases to make plain my position.

Case I.—Mrs. M., aged 42 years, a resident of Syracuse for the last four years—previous to that lived in a country village—consulted me two years ago for an acute cold in the head. and a very severe facial neuralgia, almost totally unfitting her from performing her ordinary household duties. She had been unable to eat or sleep since the advent of this attack three days previous. She had been a sufferer for about nine years and having lived in different localities, had had several different physicians, who usually saw her during an attack similar to this, and who would prescribe some opiate, large doses of which would only relieve the acuteness of the pain. She had all the teeth on right side of upper and lower jaws removed by the advice of one physician, and another recommended section of the facial nerve, but to this she would not consent.

Upon examining patient's nose, I found all the membranes intensely inflamed and swollen, the middle turbinates especially, which were fairly blue, the pressure was so great. After treating the nose with sprays and the like, a retraction of the membranes took place generally and the middle turbinates retracted in places, so that there was a space between them and the septum. The patient declared that this had given her more relief than a hypodermic of

---

1. President's address, read at the thirty-second annual meeting of the Medical Association of Central New York, at Syracuse, October 17, 1899.

morphine. I advised her to consult a specialist, which she did; had both middle turbinates removed, one of which had grown to the septum and after a period of six months, during which time she was having the operations and the mucous membranes stimulated by local treatment, she calls herself cured, and so she is, practically; yet if she takes cold she will have a slight headache for a few days until the congestion leaves the membranes.

Case II.—Mrs. W., aged 36 years, resident of a neighboring town, consulted me one year ago with the following history : She would have periodical headaches come on without warning, both frontal and occipital, so severe that she was always obliged to go to bed and send for a physician to relieve her and very often she would be in a semicomatose condition when the doctor arrived. After going through these attacks on an average of once in four or six weeks for two years, her physician told her that her case puzzled him, and advised her to go to Syracuse and consult some physician there. She came, and the friends with whom she stopped came with her to my office; she was then suffering with a dull headache over the eyes, left from an attack from which she had recently recovered. Upon examining her nose, I found a deflected septum and one middle turbinate pressing intensely. I relieved this pressure by local treatment and her headache disappeared. I advised the removal of the middle turbinate and told her I felt sure it would modify her headaches, but that there was some other cause for the severe occipital pains. She acted upon my advice and had a specialist remove the middle turbinate and felt so much relieved in three weeks, that she went home feeling sure she would never have another headache, though I tried to discourage that idea. In about eight weeks, dating from the previous attack she had another, but it was almost entirely occipital and not near so severe and did not last near so long. Her physician wrote me about it and I wrote him to advise her to return for an examination of the pelvic organs. She finally consented and I found a greatly enlarged and sensitive uterus with a bilateral laceration, to which I attributed her occipital headaches. I sent her to the hospital and after reducing the enlarged and sensitive uterus to its normal size, repaired the laceration, and in due time she went home, and, now, eight months since, informs me that she has not had a headache severe enough to prevent her from performing her household duties.

Case III.—Mrs. S., aged 65 years, resident of this city had suffered with frontal headaches and facial neuralgia of the most intense type for thirteen years. She would have spasms of pain when walking in the street, which would make her scream aloud, no matter how hard she tried to prevent it. She never passed a day or night without more or less pain. She had been led to believe by her physician that there was no relief for her except to take morphine, when the pain was too severe or when it lasted too long. This she had been doing for two years, and it was with great doubt that she sought relief in any other direction.

She consulted me eight months ago; a glance at her face indicated at once that a great amount of pain at least was due to her nose, for it was all out of shape from distended ethmoids and intranasal pres sures. A thorough examination proved this to be true. I recommended her to a specialist, who removed the middle turbinates, opened the ethmoids and secured good drainage, so that now after eight months she is greatly improved and benefited, though not entirely cured, owing to the long standing pressures and consequent weakness and unhealthy condition of the tissues, which will gradually improve by proper treatment.

In conclusion, while I do not claim any new ideas in this address, still if by it I can be the means of saving even one of these poor mortals the agony he or she must endure, I shall feel that it has been productive of much good.

